# Metabolically healthy and metabolically unhealthy obese children both have increased carotid intima-media thickness: a case control study

**DOI:** 10.1186/s12872-018-0874-5

**Published:** 2018-07-04

**Authors:** Giovanni Farello, Annarita Antenucci, Stefano Stagi, Chiara Mazzocchetti, Franco Ciocca, Alberto Verrotti

**Affiliations:** 1 Department of Pediatrics, University of L’Aquila, Via Vetoio 1, 67100 L’Aquila, Italy; 2 University of Firenze, Piazza di San Marco, 4, 50121 Firenze, FI Italy; 3 S.Savatore hospital - L’Aquila, Via Lorenzo Natali, 1, 67100 L’ Aquila AQ, Coppito Italy; 4 Department of Life, Health & Environmental Sciences, Piazzale Salvatore Tommasi 1, 67100 L’Aquila, Italy

## Abstract

**Background:**

The cardiovascular disease risk was assessed in metabolically healthy obese (MHO) children, obese children with metabolic disorders (MUO), and to a control group of normal-weight children using carotid intima-media thickness (CIMT).

**Methods:**

Participants were 204 obese children (114 M, 90 F), including 162 MUO (74 M, 88 F) and 42 MHO (24 M, 18 F), and 99 gender- and age-matched controls (45 M, 54 F). Glucose, triglycerides, total cholesterol, high-density and low-density lipoprotein cholesterol, and other serum values were determined in peripheral blood. Anthropometric parameters, blood pressure, and a carotid Doppler ultrasound scan were also acquired. The mean CIMT of obese subjects and controls was compared by analysis of variance. Abnormality of even one of the metabolic parameters assessed involved assignation to the MUO group. Mean CIMT was compared in MHO and MUO children.

**Results:**

Mean CIMT in control children was 402.97 ± 53.18 μm (left carotid artery) and 377.85 ± 52.47 μm (right carotid artery). In MHO and MUO patients CIMT was respectively 453.29 ± 62.04 and 460.17 ± 92.22 μm (left carotid artery) and 446.36 ± 49.21 and 456.30 ± 85.7 μm (right carotid artery). The mean CIMT was not significantly different in MUO and MHO children, whereas it showed a significant difference between both groups of obese children and controls (*p* < 0.01).

**Conclusion:**

CIMT was significantly greater in obese patients, also in those without metabolic alterations, than in normal-weight children. Obesity is therefore an important risk factor for cardiovascular disease in itself, also in the absence of metabolic abnormalities.

## Background

The World Health Organization (WHO) considers childhood obesity a global epidemic condition and one whose multifactorial nature hampers both therapeutic management and prevention. In 2013, 42 million children aged less than 5 years were overweight or obese [1].

Obesity plays a key role in the development of metabolic syndrome (MS) [2, 3], which in the past few decades has significantly increased among obese children [4].

However, not all the severely obese suffer from MS; indeed 10–25% of severely obese adults are “metabolically healthy” (MHO) [5–8]. Among children, the prevalence of “obesity without metabolic alterations” ranges from 4.2 to 68% according to the classification used and the reference population. A predominance of the MHO phenotype is found in females and is inversely related to age [9].

CIMT has been shown to be increased in children with traditional cardiovascular risk factors, such as obesity, hypertension, and chronic kidney disease, as compared to healthy children, Moreover [10] some longitudinal studies have shown that MS during childhood predicts the development of CVD by adulthood [11, 12] this highlight the importance of the prevention for the child obesity.

CIMT is a non-invasive measure of subclinical atherosclerosis and a good indicator of cardiovascular risk [13]. According to several studies, atherosclerosis is an early process beginning in childhood, with fatty streaks observed in carotid arteries of children and adolescents [14]. Since 2007 the European societies of cardiology and hypertension consider CIMT as a marker of organ damage [15]. The few studies that have assessed cardiovascular risk in MHO children have reported conflicting results [16–18].

The Bogalusa Heart Study, conducted in 2012, involved 1098 subjects, both children (aged 5–17 years) and adults (aged 24–43 years) showed that MHO children have favorable cardiometabolic profiles and CIMT in adulthood compared with metabolically abnormal, overweight/obese children. Further, their cardiometabolic profiles and CIMT are comparable to those of nonoverweight/obese children [19].

The STYJOBS/EDECTA study described greater CIMT and nuchal and visceral fat values in metabolically unhealthy obese (MUO) compared to MHO subjects [20]. In 2013 Reinher et al. reported similar results when they compared patients with MS to the general population [17]. In contrast, a recent study of 1617 obese subjects aged 9 to 24 years who were followed for 21–25 years found that those without metabolic alterations were at greater risk to develop type 2 diabetes mellitus and to have higher CIMT compared to normal-weight healthy subjects [21].

In order to investigate the presence of a cardiovascular risk, demonstrated through the increase of CIMT also in the metabolically healthy obese subjects, we have studied this parameter in a population of metabolically healthy obese, metabolically unhealthy obese compared to a control group.

The study aim to compare CIMT in metabolically healthy obese subjects, metabolically unhealthy obese, and normal – weight children. Additionally, we intend to establish whether the latter group of subjects may be considered to be at no risk of developing the cardiovascular complications of overweight and obesity.

## Methods

### Subjects

Participants were 204 children (114 M, 90 F) aged 6 to 16 years (mean age, 11.55 ± 2.83 years) divided into an MUO (74 M, 88 F) and an MHO (24 M, 18 F) group, and 99 controls (54 F, 45 M) whose mean age was 11.47 ± 3.63. The body mass index (BMI) of the obese children was ≥ the 95th percentile, whereas the BMI of control children was between the 25th and the 75th percentile. Partecipants were recruited at the Pediatric Clinic of the University of L’Aquila - Auxology service. Exclusion criteria were secondary obesity, other syndromes or systemic diseases, and use of medications known to alter blood pressure or lipid or glucose metabolism. We decided to study only the subject with primary obesity in order to avoid possible confounding factors: medication as metformin has been shown to exert a protective role against oxidative stress; genetic obesity (as Prader Willy syndrome) should have effect on cardiovascular system that do not depend only by obesity. For the purposes of the study, MHO participants were obese children (waist circumference ≥ 90th percentile by age and sex) without abnormal glucose/insulin ratio (HOMA – IR) or lipid metabolism compared to the reference values for age and gender and without blood pressure alterations based on the percentiles for age, gender, and height (Table 1). Obese children (waist circumference ≥ 90th percentile by age and sex) with, at least, one parameter of metabolic syndrome not in the normal range were assigned to the MUO group as established by the previous literature [22, 23].

**Table 1:** Characteristics of MHO patients

WC ≥ 90th percentile by age and sex [34]	
HOMA-IR < 75th percentile by age and sex [35]	
Triglycerides<95th percentile by age and sex [36]	
HDL-C > 5th percentile by age and sex [36]	
PAS and PAD <90th percentile by age, sex and height [37]	

### Anthropometric measurements

Standing height was measured with a HOLTAIN stadiometer to the nearest 0.1 cm. Weight (to the nearest 0.1 kg) and body composition (trunk and total fat and lean mass) were measured with a TANITA BC-418 MA bioimpedance analyzer. BMI was calculated using the formula BMI = kg/m^2^. BMI for age was expressed as BMI-SDS based on age- and gender-specific percentiles using the 2006 Italian growth charts [24].

Waist circumference (WC) was measured at the highest point of the iliac crest at the end of a normal expiration [25]. The WC percentile was calculated according to the table of the IDF consensus worldwide definition of MS in children and adolescents for the European population. The waist-to-height ratio was also calculated [26].

Blood pressure was measured in sitting position using a mercury sphygmomanometer after a 10 min rest. Measurements were taken from the upper arm with an appropriate sized cuff. Systolic (SBP) and diastolic (DBP) blood pressure were read to the nearest 2 mmHg and recorded at the appearance and disappearance of Korotkoff’s sounds, respectively. The lowest of 3 consecutive readings was recorded.

### Blood samples

With the written informed consent of the parents, blood was drawn from the cubital vein of each participant (in sitting position between 8.00 and 10.00 a.m.) after 12 h fasting to determine: fasting plasma glucose (FPG) and insulin (FPI), serum triglycerides (TG), total cholesterol, high-density lipoprotein cholesterol (HDL-C), low-density lipoprotein cholesterol (LDL-C), 25-hydroxyvitamin D, alanine aminotransferase (ALT), aspartate aminotransferase (AST), and gamma-glutamyl transpeptidase (ƔGT).

Plasma glucose, insulin, triglycerides, HDL-C, AST, ALT, and ƔGT, were analyzed with an ARCHITECT apparatus (Abbott).

Glucose and insulin levels were used to estimate basal insulin resistance (IR) by the homeostatic model assessment (HOMA-IR): fasting blood glucose (mg/dl) x fasting insulin (mg/dl)/405 [27].

CIMT was measured with color Doppler ultrasound by a vascular surgeon (F.C.) using a Technos MPX Diagnostic Ultrasound machine (Esaote). Patients lay supine with the head slightly tilted contralateral to the side being examined; shoulder elevation allowed stretching the neck of subjects with a short neck. The far wall of the left and right common carotid artery was scanned 1 cm before the carotid bulb over a length of 1 cm [18]. CIMT was measured by the semiautomatic instrument, which averaged 6 values from each artery and provided the result in micrometers.

### Data processing and statistical analysis

An estimated sample size of 300 subjects, was calculated to be adequate to achieve a 95% power to detect a moderate effect size (Cohen’s f: 0.25) with 2 df and an α of 0.05 on CIMT. Power analysis was performend by G*Power version 3.1.9.2 [28].

The mean CIMT values of MUO, MHO, and control subjects were compared by ANOVA.

Data entry and analysis were performed using the Statgraphics–Centurion Ver XV statistical package. Results are presented as mean ± standard deviation (SD). Differences in the means of the variables were tested by ANOVA. Data distribution was tested for normality using the Shapiro-Wilk test. A post-hoc Fisher LSD analysis was performed using the independent “t” test in case of normally distributed continuous variables. Probability values < 0.05 were considered statistically significant.

## Results

The mean values and SD of the anthropometric and metabolic parameters determined in MHO, MUO, and control children are summarized in Table 2.

**Table 2:** Mean values and SD of the anthropometric and metabolic parameters determined in MHO, MUO, and control children

	MHO patients	MUO patients	Controls
Age	11.32 ± 2.89	11.26 ± 2.73	11.47 ± 3.63
Weight (kg)	59.38 ± 10.98*	60.60 ± 20.13*	39.21 ± 4.32**
Height (cm)	145.27 ± 8.75	143.87 ± 15.69	142.4 ± 9.34
BMI	27.35 ± 2.12*	28.44 ± 4.14*	17.8 ± 2.56**
BMI-SDS	1.93 ± 0.2*	2.16 ± 0.39*	0.43 ± 0.11**
WC (cm)	85.85 ± 7.9*	84.4 ± 11.3*	58.1 ± 5.6**
WC/h	0.59 ± 0.09*	0.59 ± 0.04*	0.46 ± 0.03**
Triglycerides (mg/dl)	60.5 ± 18.5*	92.84 ± 48.89**	63.4 ± 21.6***
HDL-C (mg/dl)	52 ± 5.79*	39.09 ± 8.99**	59.3 ± 6.21***
LDL-C (mg/dl)	89.57 ± 23.4	88.04 ± 24.55	87 .34 ± 19.5
SBP (mm Hg)	114.17 ± 4.69*	118.37 ± 13.97*	100.12 ± 5.23**
DBP (mm Hg)	69.58 ± 6.2*	77.45 ± 8.96*	65.54 ± 3.56**
Glycemia (mg/dl)	78.14 ± 5.67	77.01 ± 6.91	76.03 ± 7.34
Insulin	8.36 ± 1.96	9.17 ± 5.74	6.34 ± 1.76

* vs **p < 0.01

* vs ***p = not significant

The mean CIMT values of MHO, MUO, and control subjects are reported in Table 3.

**Table 3:** Mean CIMT values in control subjects and MUO and MHO patients

	MHO patients	MUO patients	Controls
Left CIMT (μm)	453.29 ± 62.04**	460.17 ± 92.22**	402.97 ± 53.18*
Right CIMT (μm)	446.36 ± 49.21**	456.30 ± 85.7**	377.85 ± 52.47*

* vs **p < 0.01

**Fig. 1:**
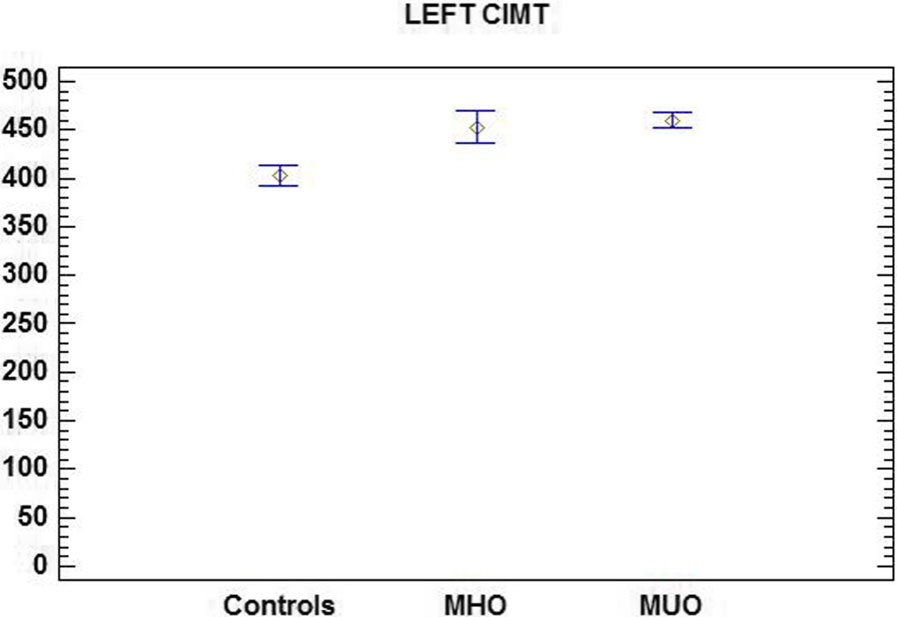
Mean left CIMT in control subjects and MUO and MHO patients

**Fig. 2:**
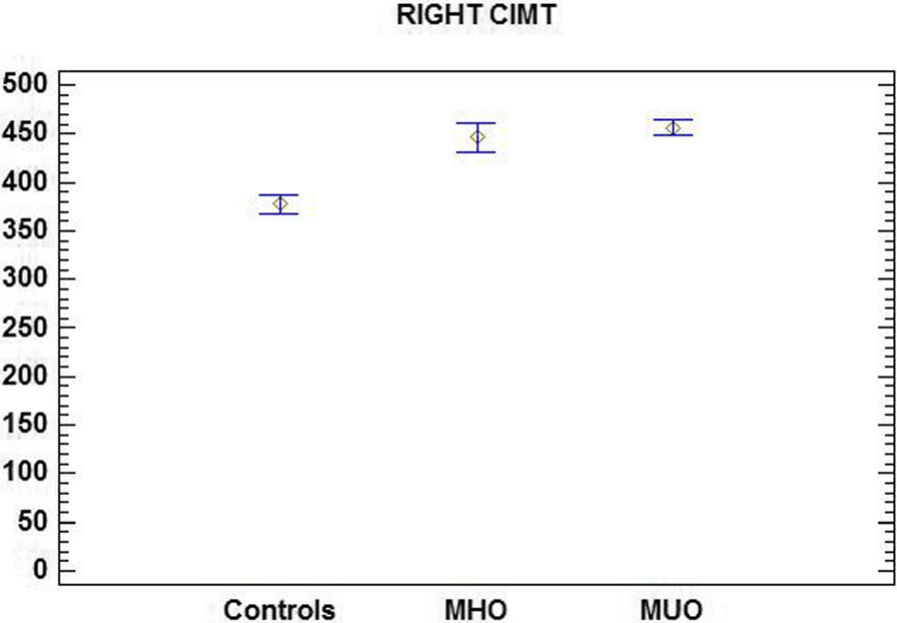
Mean right CIMT values in control subjects and MUO and MHO patients

Comparison of mean CIMT highlighted a statistically significant difference (*p* < 0.01) between the groups of obese children (MHO and MUO) and controls for both the left and the right carotid artery (Figs. 1 and 2).

No associations emerged between CIMT and total cholesterol, LDL-C, and triglyceride levels.

## Discussion and conclusions

In our study we confirmed higher values of CIMT in MHO and MUO children respect control, non obese, subjects.

Similar results are reported by Koskinen et al. [21] showing that isolated obesity represent a cardiovascular risk factor, Urbina et al. [10] stated the abnormalities in carotid thickness and stiffness in youth with obesity and T2DM. These results in youth put in evidence the need to prevent and to treat wherever possible overweight and obesity.

Several studies have found that CIMT is associated with an increased risk of atherosclerosis and later myocardial infarction and stroke in adults [29–31].

We did not find difference in the value of CIMT in MHO and MUO subjects, and both group showed a CIMT statistically higher respect normal weight subjects. This result is in contrast with previous study that, however, had a case study of obese adults [32].

The possible explanation of these controversial results can be due to, different methods used to measure CIMT. Guidelines on the method of conducting the color Doppler ultrasound examination of the carotid arteries for CIMT evaluation have been drafted to address the problem only recently [18] and in our study CIMT was measured according to these guidelines.

The CIMT values measured in the present study were higher, with statistical significance, in both MUO and MHO subjects respect control subjects. This finding highlights that in our casuistric that obesity, even in the absence of alterations in metabolic parameters (MHO) increases the risk for cardiovascular disease.

This is a cross-sectional study and this can represent a limitation because it was not possible to assess at which age the different groups start to develop obesity: we cannot exclude that the MHO group consisted of subjects where obesity had come up later.

A pilot study among obese adolescents showed that an earlier onset and a longer duration of obesity was associated with unhealthy metabolic characteristics in boys and not in girls [33], this work, although in a limited casuistric, highlight the need to study the age of onset of obesity and correlate it with the presence of metabolic alterations.

Another limitation of our study is that we have not studied obese with documented metabolic syndrome; moreover, in the MUO group we did not subdivide subjects with only one metabolic alteration compared to those with two or more metabolic alterations.

The strength of our study is to have shown an increase in CIMT regardless the presence of metabolic alterations in the obese child.

Recent studies highlight the need to investigate the impact of gene expression and genetic profile as well as metabolic pathways, such as role of free fatty acids, insulin signaling pathway in skeletal muscle and liver in MHO compared to metabolically non-healthy obese individual to improve our understanding of the pathophysiology underlying the development of insulin resistence and metabolic morbidity in the non – MHO patients [7].

The present data confirm the existence of a subgroup of obese children without metabolic abnormalities. Moreover, although the latter patients are probably at lower risk of developing MS than obese patients with early metabolic alterations, they do not seem to be at lower risk of developing cardiovascular disease as young adults, since their CIMT was significantly greater than that of normal-weight controls.

In conclusion, this study underscores the fact that even in metabolically healthy obese children is present higher value of CIMT and this cardiovascular risk factor require close clinical monitoring exactly like obese children with metabolic abnormalities; this includes color Doppler ultrasound examination performed according to the latest AEPC guidelines besides a suitable dietary and behavioral program to correct the weight excess and promote the stable achievement of normal weight over time.

## Abbreviations

BMI: Body mass index
CIMT: Carotid Artery Intimamedia Thickness
CVD: Cardiovascular disease
MHO: Metabolically healthy obese
MUO: Metabolically unhealthy obese
WC: Waist circumference
